# Engineering Bacteria and Their Derivatives for Cancer Immunotherapy

**DOI:** 10.34133/bmef.0047

**Published:** 2024-06-28

**Authors:** Yuji Tang, Chen Yu, Lang Rao

**Affiliations:** ^1^ Institute of Biomedical Health Technology and Engineering, Shenzhen Bay Laboratory, Shenzhen 518132, China.; ^2^ State Key Laboratory of Crop Stress Biology for Arid Areas, College of Life Sciences, Biomass Energy Center for Arid and Semi-Arid Lands, Northwest A&F University, Yangling, Shaanxi 712100, China.; ^3^ Institute of Cancer Research, Shenzhen Bay Laboratory, Shenzhen 518132, China.

## Abstract

Leveraging bacteria for cancer immunotherapy has gradually attracted wide attention since the discovery of “Cloey’s toxin.” However, one of the persistent challenges for bacteria-based therapy is striking a balance between safety and immunogenicity. Genetically engineered bacteria with virulence factors removed could further enhance antitumor ability by integrating genetic elements. In addition, bacterial derivatives, including outer membrane vesicles (OMVs) produced by bacterial secretion and nanovesicles synthesized by modification of OMVs, could enhance antitumor immunity while improving safety. This perspective discusses the unique advantages of engineered bacteria and their derivatives for immunotherapy, as well as the challenges that need to be overcome to achieve clinical translation.

Before the 19th century, there were recorded cases of sarcoma patients whose tumors miraculously shrank or disappeared after being infected with erysipelas. In 1899, William Coley used Coley’s toxin, a bacterial-derived drug, to treat sarcoma patients. This marks the beginning of tumor immunotherapy. Until the drug was discontinued, its mechanism of action remained unclear [1]. Bacteria-based immunotherapy, such as the Bacillus Calmette-Guerin (BCG) vaccine made from live *Mycobacterium tuberculosis*, effectively treats cancer and reduces recurrence rates [2]. However, high toxicity of drugs made from these bacteria hinders their widespread use in clinical therapy [3]. Nowadays, the bacteria can be turned into therapeutic vectors with adjustable virulence, reproductive capacity, and immunostimulatory properties for clinical applications through gene technology. Moreover, bacterial derivatives can inherit the immunostimulatory capacity of genetically engineered bacteria and reduce the therapeutic risk because of the lack of reproductive capacity [4]. Considering the long-term symbiotic relationship between bacteria and the human body, bacteria and their derivatives have good tumor tropism, tumor immune activation ability, and biocompatibility, thus being suitable for clinical practice of treatment.

Initially, genetically engineered bacteria remain attenuated only by removing virulence factors, which are not therapeutically effective in clinical practice. The therapeutic efficacy of attenuated bacteria is limited by its inadequate tumor-targeting ability and surrounding area damage during proliferation. To address this issue, tumor-targeting ability and proliferation ability of bacteria should be regulated. It can be accomplished by the bacteria themselves or induced by external factors. For instance, the integration of high-affinity CD47nb into the synchronized lysis circuit (eSLC) allows bacteria to colonize at tumor sites and release CD47nb locally, targeting tumor cells expressing CD47 (Fig. 1A) [5]. Alternatively, bacteria can be designed as sensing circuits in response to the tumor signals using inducible promoters [6]. Binding the sensing circuits through “AND” logic gates, the bacteria can be induced to express functional proteins and lysed in the tumor to activate immune cells [7,8]. The targeting and proliferating properties of bacteria can also be regulated by genetically engineered methods. The targeting and proliferating properties of bacteria can also be regulated by genetically engineered methods, such as the use of auxotrophic bacteria that can selectively grow in tumors and the modification of tumor-associated antigens [9].

**Fig. 1.**
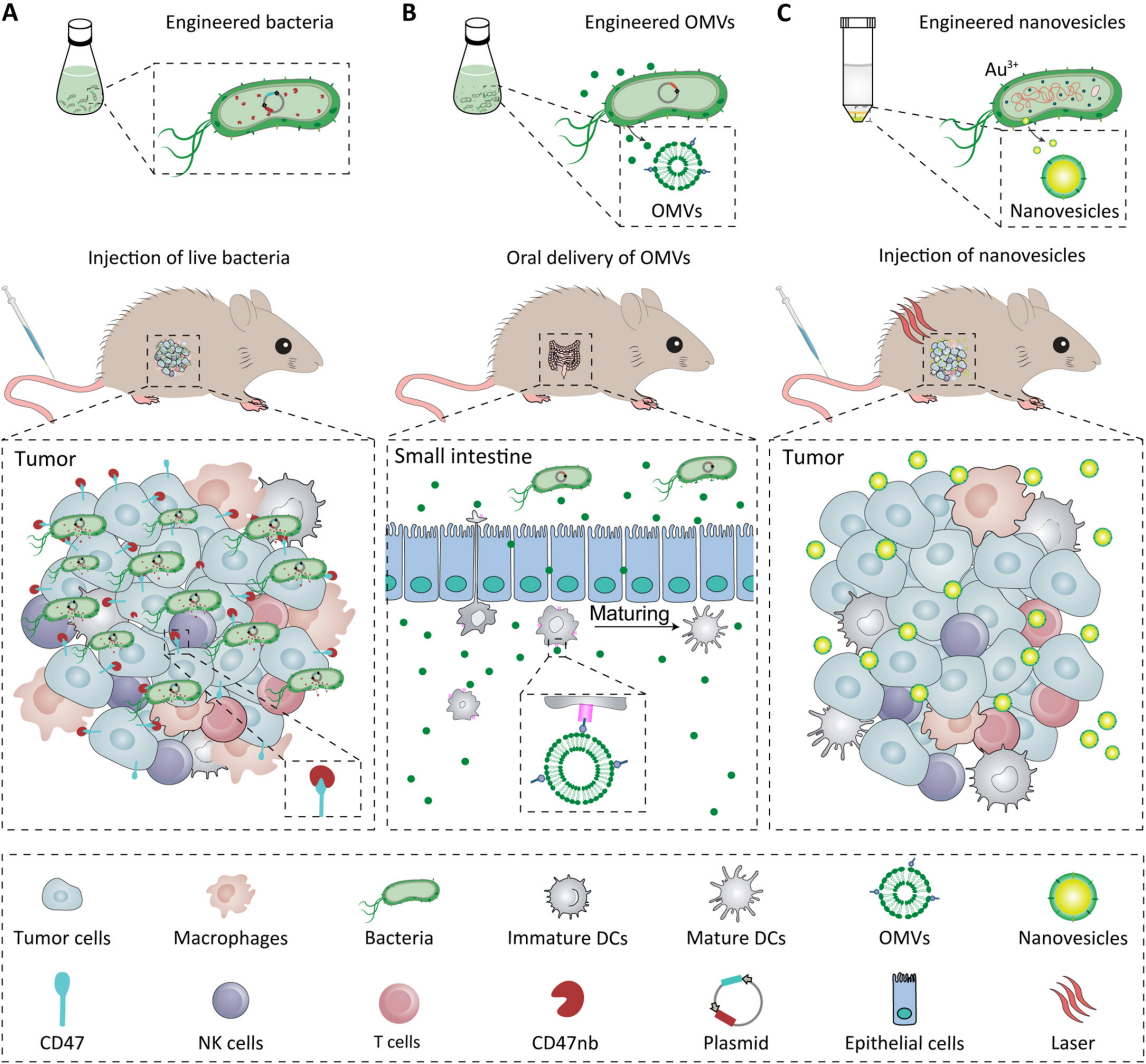
Engineering bacteria and their derivatives for cancer immunotherapy. (A) Genetically engineered bacteria release antibodies by intratumoral quorum lysis. Activation and proliferation of tumor-infiltrating lymphocytes induces durable and systemic antitumor immunity. (B) Genetically engineered OMVs cross small intestinal epithelial cell walls to present tumor antigens to immature dendritic cells (DCs), which effectively activate tumor antigen-specific immune responses. (C) Genetically engineered NVs lysed tumor cells under laser irradiation to release tumor antigens, which costimulate immune cells with bacterial components. They elevate baseline immunity and amplify drug-induced immune responses.

In addition to bacteria as a whole as the main body of treatment, outer membrane vesicles (OMVs) have emerged as an alternative to bacteria. OMVs secreted by Gram-negative bacteria retain the ability to stimulate innate immunity by inheriting various pathogen-associated molecular patterns [10]. It is impossible for OMVs to reproduce, which can significantly reduce the risks of bacteria-based therapy. Recent studies have shown that OMVs can accumulate in lymph nodes due to their small size [11]. Their ability to be produced in large quantities by bacterial fermentation makes OMVs attractive candidate vaccine vectors. Intraperitoneal administration of OMVs increases the production of antigen-presenting cell progenitors and the activation of tumor antigen-specific T cells [12,13]. Moreover, the use of engineered OMVs with antigens to penetrate the intestinal epithelial barrier and interact with immune cells has overcome long-standing technical barriers to oral vaccines (Fig. 1B) [14]. However, the adoption of engineered OMVs always faces dosing challenges, and excessively high doses of OMVs can lead to the overactivation of systemic immunity.

To overcome the current limitations of using engineered OMVs, it is possible to use nanovesicles (NVs), which are prepared by modifying naturally occurring OMVs. NVs are endowed with multifunctional properties to stimulate a comprehensive and selective immune response and shield from some of the risks of applying OMVs. Membrane hybridization is a technique that confers membrane properties to hybridized membrane-derived cells [15]. One strategy is to use bacteria cytoplasmic membranes and autologous tumor cell membranes to generate hybrid NVs that deliver antigens and adjuvants to antigen-presenting cells [16]. This hybridization strategy reduces the risk of cytokine storm and sepsis due to the dilution of various bacterial-derived components by autologous cell membranes. The systemic inflammation triggered by OMVs can also be overcome by using biomineralization strategies. OMVs are encapsulated with pH-sensitive calcium phosphate nanoshells for delivery to tumor tissues [17]. Moreover, the NVs generated by bacterial biomineralization also trigger local thermotherapy to enhance tissue infiltration by effector immune cells (Fig. 1C) [18]. In addition, direct wrapping with a polyethylene glycol (PEG) layer containing diselenide bonds can achieve the same purpose [19]. Disruption of the PEG by localized radiation therapy exposes the immunostimulatory bacterial components. With the above shielding or dilution, the drug’s side effects are mitigated at a dose that ensures efficacy. The therapeutic effect on the tumor lesion area is enhanced by other modifications on the substance. Thus, NVs can overcome the dosage challenges faced by natural OMVs as a mild but therapeutically effective biomaterial.

Bacteria and their derivatives have great potential in biomedical applications. Due to strong genetic engineering potential of bacteria, similar integration of eSLC gene modification approaches could better control bacteria to exert antitumor effects [20]. The application of genetically engineered bacteria can also be carried over to its derivatives. OMVs and NVs, as bacterial derivatives, can also be used to enhance their therapeutic efficacy by overcoming dosage issues through other approaches. Recent studies have found that killing tumor-associated bacteria can provide new antigens for cancer immunotherapy [21]. Subsequently, NVs made directly from symbiotic bacteria within tumors will also broaden the research direction of antitumor immunotherapy [22,23]. This immune mobilization strategy using bacteria and their derivatives to alter the underlying innate immune function is a promising approach to active immunotherapy.
